# Mendelian Randomization Assessment of the Genetic Effects of Lipid‐Lowering Drugs on Digestive System Cancers

**DOI:** 10.1002/fsn3.70293

**Published:** 2025-05-19

**Authors:** Keru Ma, Hao Wang, Yubo Du, Tianyu Chen, Dongxu Yang, Yue Li, Dalin Li

**Affiliations:** ^1^ Department of Breast Surgery Harbin Medical University Cancer Hospital Harbin China; ^2^ Department of Medical Oncology Harbin Medical University Cancer Hospital Harbin China

**Keywords:** digestive system cancer, eQTL, GWAS, LDL, mendelian randomization

## Abstract

The relationship between lipid‐lowering drugs and the risk of digestive system cancers remains unclear. This study aims to assess the risk association between lipid‐lowering drugs and digestive system cancers through mendelian randomization (MR) analysis. We utilized genetic instruments to substitute for the exposure to lipid‐lowering drugs, including expression quantitative trait loci (eQTL) for HMGCR, PCSK9, and NPC1L1, as well as genetic variants associated with low‐density lipoprotein (LDL) from the Global Lipids Genetics Consortium's genome‐wide association study (GWAS) data for target genes. We used MR and SMR methods to assess the risk estimates of lipid‐lowering drug target genes on digestive system tumors. The MR analysis indicated a negative association between HMGCR‐mediated LDL and hepatocellular carcinoma (OR = 0.06, 95% CI: 0.00–0.81, *p* = 0.03), and a positive association between NPC1L1‐mediated LDL and gastric cancer risk (OR = 15.45, 95% CI: 5.96–40.56, *p* < 0.01). In the SMR analysis, it was observed that HMGCR expression decreased the risk of hepatocellular carcinoma (OR = 0.11, 95% CI: 0.02–0.68, *p* = 0.02), while NPC1L1 expression increased the risk of gastric cancer (OR = 1.33, 95% CI: 1.08–1.64, *p* < 0.01). Our study results suggested a potential risk association between HMGCR inhibitors and NPC1L1 with hepatocellular carcinoma and gastric cancer.

## Graphical abstract

Mendelian randomization reveals the risk association between lipid‐lowering drugs and digestive tract cancers; HMGCR inhibitors and NPC1L1 may be potentially associated with the risk of hepatocellular carcinoma and gastric cancer; This study provides some evidence for the clinical application of lipid‐lowering drugs. CRC, colorectal cancer; EC, esophageal cancer; GBC, gallbladder cancer; GC, gastric cancer; HEIDI: Heterogeneity in Dependent Instruments; LC, liver cancer; LDL, low‐density lipoprotein; PC, pancreatic cancer; PPH4, posterior probability of H4; QTL, quantitative trait loci; SMR, summary‐based mendelian randomization.

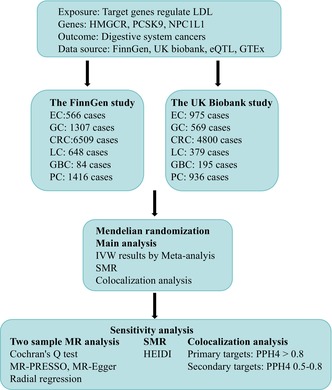

## 1 Introduction

In 2018, cancers of the digestive system were responsible for over 26% of global cancer cases and accounted for more than 35% of cancer‐related fatalities, resulting in approximately 4.8 million new diagnoses and 3.4 million deaths (Arnold et al. 2020). Risk factors for digestive cancers have been extensively studied and shared to some extent (Ulrich et al. 2018). Preventive measures that target risk factors may help prevent and control digestive system cancers (Arnold et al. 2020), but effective drug treatments are still lacking. Therefore, identifying effective therapeutic agents may reduce the social burden of digestive system cancer.

The existing studies indicate that lipid plays a crucial role in digestive system cancers (Liu et al. 2022; Vriens et al. 2019). Possible mechanisms include influencing the biological energy of tumor cells, membrane biosynthesis, and intracellular signal transduction (Xiao and Zhou 2017; Yang et al. 2022). Epidemiological studies have shown that lipid‐lowering drugs have a preventive effect on digestive system cancers (Poynter et al. 2005; Demierre et al. 2005). However, current studies present contradictory results (Coogan et al. 2007; Jacobs et al. 2006). There are certain methodological limitations and residual confounding in some studies, which result in the incomplete establishment of a definitive causal relationship between lipid‐lowering drugs and digestive system cancers.

HMG‐CoA reductase inhibitors (HMGCR), commonly known as statins, represent the most prevalent class of lipid‐lowering drugs that have the advantage of high safety and low cost (Wang et al. 2018). Proprotein convertase subtilisin/kexin type 9 (PCSK9) and Niemann–Pick C1‐Like 1 (NPC1L1) are novel targets for lipid‐lowering drugs, playing a crucial role in regulating circulating low‐density lipoprotein levels, and both have received Food and Drug Administration approval (Sabatine 2019; Williams et al. 2020). Some observational studies have identified potential therapeutic benefits of the aforementioned lipid metabolism targets for digestive system cancers (Alannan et al. 2022; Kwon et al. 2021). However, randomized controlled trials (RCTs) remain the gold standard for determining drug efficacy, and currently, there is a lack of large‐scale RCTs. Invasive tissue biopsies remain the gold standard in clinical trials, but it is difficult to dynamically monitor individuals. Meanwhile, due to confounding factors, it remains unclear whether lipid‐lowering drugs are effective in digestive system cancers.

Mendelian randomization (MR) is based on genome‐wide association studies (GWAS) for causal inferences about exposure and outcome. Genetic variants are randomly assigned at birth; MR minimizes confounding bias and avoids reverse causation. For drug‐target MR, genetic variations within genes encoding protein targets can affect the expression of the target gene, which can predict the outcome of RCTs and ultimately elucidate the causal inference of the drug‐gene target on the outcome (Gill et al. 2019).

Hence, we used GWAS summary statistics for digestive system cancers, including esophageal cancer (EC), gastric cancer (GC), colorectal cancer (CRC), liver cancer (LC), pancreatic cancer (PC), and gallbladder cancer (GBC). Through drug‐target MR analysis, we investigated the causal relationships between genetically predicted inhibitors of HMGCR, PCSK9, and NPC1L1 and the above diseases.

## 2 Materials and Methods

This study was conducted in accordance with the Strengthening the Reporting of Observational Studies in Epidemiology guidelines (STROBE, Supplementary STROBE‐MR checklist table) (https://www.strobe‐mr.org/). As this study involved re‐analyzing previously collected and published data, no further ethics approval was required.

### 2.1 Study Produce

**FIGURE 1:**
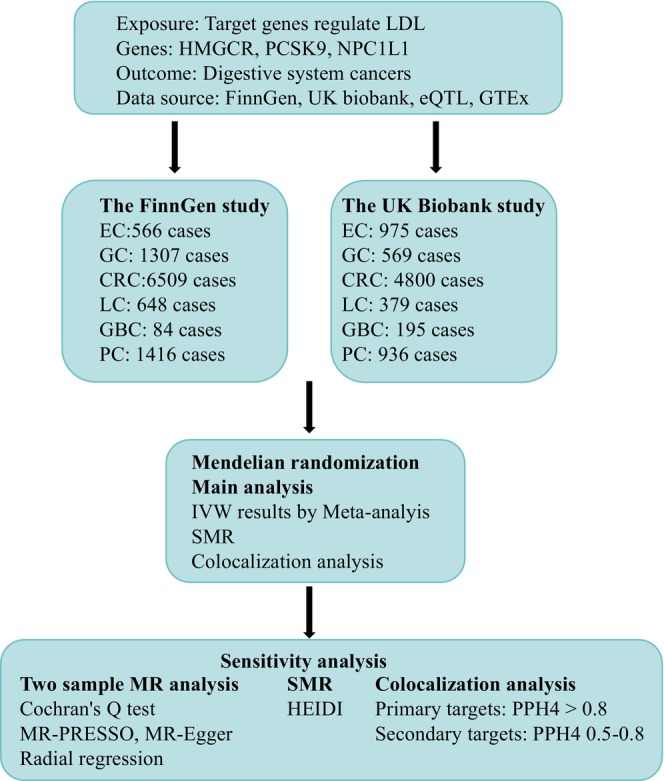
Study design. CRC, colorectal cancer; EC, esophageal cancer; GBC, gallbladder cancer; GC, gastric cancer; HEIDI, Heterogeneity in Dependent Instruments; LC, liver cancer; LDL, low‐density lipoprotein; PC, pancreatic cancer; PPH4, posterior probability of H4; QTL, quantitative trait loci; SMR, summary‐based mendelian randomization.

The study workflow is illustrated in Figure 1. Data were sourced from GWAS studies and summary data from expression quantitative trait loci (eQTL) studies (Table S1). The research process was based on the three major assumptions of MR: (1) a significant association between genetic variation and exposure (the correlation hypothesis); (2) genetic variation is unrelated to confounding factors (exclusivity hypothesis); and (3) genetic variation influences outcomes only through exposure (independence hypothesis).

### 2.2 Selection of Genetic Instruments

We used single nucleotide polymorphisms (SNPs) within the 100 kb window of the drug target genes, namely HMGCR, PCSK9, and NPC1L1, which are significantly associated with LDL levels in the genome‐wide context (*p* < 5 × 10^−8^). The summary GWAS data for LDL came from the Global Lipids Genetics Consortium (GLGC) (*N* = 173,082) (Willer et al. 2013). To minimize multicollinearity interference, the SNPs used as a tool aggregate with each other to the linkage disequilibrium (LD) threshold *r* ^2^ < 0.6, which will contain more relevant variants, improve precision/power, and prevent false *p*‐values, the threshold that has been well explored in previous lipid metabolism targets study (Schmidt et al. 2020). Additionally, LDL was a well‐recognized risk factor for coronary artery disease, and previous studies had extensively demonstrated that elevated LDL in GLGC significantly increased the risk of coronary artery disease (Li et al. 2023; Xie et al. 2023). This provided strong evidence for the strength of the current instrumental variables and the validity of the data; therefore, it will not be discussed in detail in this article.

### 2.3 GWAS Data for Outcomes

The GWAS summary data for seven digestive system cancers, esophageal cancer, gastric cancer, colorectal cancer, liver cancer, gallbladder cancer, and pancreatic cancer, were obtained from the UK Biobank study, the FinnGen study (Kurki et al. 2023), and the pan‐ancestry genetic analysis of the UK Biobank (pan‐UKB, https://pan.ukbb.broadinstitute.org/). The above GWAS data were classified into discovery cohorts and replication cohorts (Table S1). The UK Biobank was a large open database that enrolled approximately 500,000 adults from 2006 to 2010. Pan‐UK Biobank conducted a multi‐ancestry analysis of 7228 phenotypes using a generalized mixed‐model association testing framework, which included 16,131 genome‐wide association studies. The analyses were adjusted for age, sex, age × sex, age^2^, age^2^ × sex, and the top 10 principal components (Bycroft et al. 2018). We extracted 566 EC, 1307 GC, 6509 CRC, 648 LC, 84 GBC, and 1416 PC in Finn Gen, and extracted 975 EC, 569 GC, 4800 CRC, 379 LC, 195 GBC, and 936 PC in UK Biobank. The estimated results from Finn Gen and UK Biobank were combined using a meta‐analysis approach. In addition, we performed additional subgroup analysis to reveal as much as possible the effects of lipid‐lowering drugs on digestive system cancers, including esophageal adenocarcinoma, lower third of esophageal cancer, cardia gastric cancer, colon cancer, rectal cancer, hepatocellular carcinoma, intrahepatic cholangiocarcinoma, and pancreatic head cancer; all subgroup data were derived from UK Biobank, Finn Gen, and pan‐UK Biobank. For the available public data, we performed repeated analyses to ensure the robustness of the results.

### 2.4 SMR Analysis

We used expression quantitative trait loci (eQTL) as instruments; we used the summary data‐based MR (SMR) (https://cnsgenomics.com/software/smr/) method to evaluate the effect estimates of target genes on outcomes (Zhu et al. 2016). Summary data for different tissues were obtained from the eQTLs summary data of the eQTL Gen consortium and the Genotype‐Tissue Expression Project (GTEx‐v8) (Võsa 2018; GTEx Consortium 2017). We adopted the default settings of SMR and used the cis‐eQTL generation genetic tool to define eQTLs within 1 mb on either side of the coding gene. We identified eQTL SNPs significantly associated with the expression of HMGCR and PCSK9 in blood and the expression of NPC1L1 in subcutaneous adipose tissue (minor allele frequency [MAF] > 0.1, *p* < 5 × 10^−8^). There are no eQTLs at a significant level available for NPC1L1 in blood or other tissues. We removed SNPs in high LD with the top eQTL (*r* ^2^ > 0.9) and SNPs in low LD with the bottom eQTL (*r* ^2^ < 0.05). The Heterogeneity in Dependent Instruments (HEIDI) test was conducted to assess the null hypothesis of a single causal variable association. If the result of the HEIDI test was less than 0.05, it indicated the presence of heterogeneity in the current findings. For the replication cohorts, we still used SMR for replicate analyses.

### 2.5 Sensitivity Analysis

We used the *F*‐statistic to assess the strength of instrumental variables, with an *F*‐value greater than 10 indicating strong predictive capability of the instrumental variables (Pierce et al. 2011). We used inverse variance weighting (IVW), MR‐Egger, weighted median, and weighted mode to assess the causal association of genetically mediated lipid‐lowering treatment with digestive system cancers. Among these, IVW (fixed effects/random effects) served as the primary result in the MR analysis (Burgess et al. 2017). The Cochran's *Q* test was used to assess the heterogeneity of SNPs, and the Cochran's *Q* test less than 0.05 indicated the presence of heterogeneity (Burgess et al. 2013). We used MR‐Egger intercept and pleiotropic residuals and outliers (MR‐PRESSO) to assess horizontal pleiotropy, and the *p*‐value less than 0.05 indicated the presence of horizontal pleiotropy (Hemani et al. 2018). MR‐PRESSO was used to correct for the influence of outliers on MR results (Verbanck et al. 2018). Furthermore, radial plots and radial regression were used to further reduce the effect of horizontal pleiotropy on MR results (Bowden et al. 2018). After excluding outliers with horizontal pleiotropy, we repeated the MR analysis on the discovery and replication cohorts. Considering multiple testing, we applied Bonferroni correction to adjust the significance level threshold, considering *p* < 0.02 (0.05/3) as strong evidence and 0.02 ≤ *p* < 0.05 as suggestive evidence. In addition, since a single SNP may be associated with the expression of multiple genes, this may lead to the presence of horizontal pleiotropy. We identified genes near the top SNP (within a 1mb window) and examined whether the expression of these genes was associated with genetic variation to assess the risk of pleiotropy. SMR analysis was then conducted to check whether these genes were associated with outcomes.

### 2.6 Colocalization Analysis

We conducted colocalization analysis using the coloc R package (Giambartolomei et al. 2014) to determine whether there is a direct association between drug targets and outcomes. In colocalization analysis, Bayesian methods evaluate the following five exclusive hypotheses for each locus: (1) not associated with any trait; (2) exclusively associated with trait 1; (3) exclusively associated with trait 2; (4) associated with both traits but driven by different SNPs; (5) associated with both traits and driven by the same causal variant. The current analysis provided posterior probabilities for each hypothesis test (H0, H1, H2, H3, and H4). We pooled different tissues from eQTL Gen and GTEx data to determine whether the target genes share the same locus variation with outcomes (25), including blood, subcutaneous fat, visceral omentum fat, esophageal, stomach, colon, liver, and pancreas. In addition, for the GWAS data on LDL, we further selected loci within a 50 kb range of the target genes for colocalization analysis with outcomes (Venkateswaran et al. 2018). If there is no genetic sharing between the two phenotypes, it can further strengthen the current positive MR results. Finally, based on the previous study, genes with the highest evidence of colocalization (PPH4 > 0.8) were considered primary targets; genes with moderate evidence of colocalization (0.5 < PPH4 < 0.8) were considered secondary targets; the remaining results are considered tertiary targets (Chen et al. 2023).

All the statistical analyses mentioned above were conducted using R version 4.3.1.

## 3 Results

### 3.1 Genetic Tools and Digestive System Cancers

We identified 13 SNPs as genetic tools in HMGCR, 22 SNPs in PCSK9 as genetic tools and 5 SNPs in NPC1L1 as genetic tools (Table S2). The *F*‐statistics were all greater than 30, indicating that instrumental variable bias was less likely to have impacted the analysis. Table S3 shows the relevance of all drug targets to digestive system cancers. In the discovery cohort, IVW analysis revealed a negative correlation between LDL mediated by HMGCR and GC (OR = 0.46, 95% CI: 0.30–0.72, *p* < 0.01) and PC (OR = 0.45, 95% CI: 0.29–0.69, *p* < 0.01). LDL mediated by PCSK9 was negatively associated with EC (OR = 0.61, 95% CI: 0.39–0.94, *p* = 0.03), GC (OR = 0.71, 95% CI: 0.53–0.94, *p* = 0.02), and LC (OR = 0.64, 95% CI: 0.42–0.96, *p* = 0.03). LDL mediated by NPC1L1 showed a positive correlation with EC (OR = 6.50, 95% CI: 1.34–31.42, *p* = 0.02), GC (OR = 20.17, 95% CI: 7.23–56.30, *p* < 0.01), CRC (OR = 2.64, 95% CI: 1.64–4.26, *p* < 0.01), and liver cancer (OR = 9.25, 95% CI: 2.11–40.51, *p* < 0.01). In the replication cohort, the results for HMGCR and NPC1L1 were not confirmed. However, the trend for PCSK9 was consistent with the discovery cohort. PCSK9‐mediated LDL was negatively associated with EC (OR = 0.65, 95% CI: 0.43–0.98, *p* = 0.04), GC (OR = 0.39, 95% CI: 0.23–0.67, *p* < 0.01), and LC (OR = 0.57, 95% CI: 0.41–0.78, *p* < 0.01) (Figures S1–S3).

In the meta‐analysis, HMGCR‐mediated LDL was negatively associated with PC (OR = 0.51, 95% CI: 0.36–0.71, *p* < 0.01) (Figure S1). PCSK9‐mediated LDL was negatively associated with EC (OR = 0.63, 95% CI: 0.47–0.85, *p* < 0.01), GC (OR = 0.55, 95% CI: 0.31–0.98, *p* = 0.04), and LC (OR = 0.59, 95% CI: 0.46–0.76, *p* < 0.01) (Figure S2). NPC1L1‐mediated LDL was positively associated with GC (OR = 15.54, 95% CI: 5.96–40.56, *p* < 0.01) and CRC (OR = 2.37, 95% CI: 1.60–3.53, *p* < 0.01) (Figure S3).

### 3.2 Genetic Tools and Digestive System Cancer Subsites

Tables S3 and S4 show the correlation of all drug targets with digestive system cancers. In the subgroup analysis, for the replicable subgroups in the discovery cohort, the analysis revealed that HMGCR‐mediated LDL was negatively associated with rectal cancer (OR = 0.61, 95% CI: 0.46–0.80, *p* < 0.01) and hepatocellular carcinoma (OR = 0.21, 95% CI: 0.08–0.59, *p* < 0.01). Additionally, PCSK9‐mediated LDL was negatively associated with rectal cancer (OR = 0.60, 95% CI: 0.48–0.75, *p* < 0.01), while NPC1L1‐mediated LDL was positively associated with rectal cancer (OR = 4.57, 95% CI: 2.10–9.95, *p* < 0.01). For the replication cohort, HMGCR‐mediated LDL was negatively associated with hepatocellular carcinoma (OR = 0.01, 95% CI: 0.00–0.05, *p* < 0.01). PCSK9‐mediated LDL was negatively associated with rectal cancer (OR = 0.70, 95% CI: 0.52–0.96, *p* = 0.03). NPC1L1‐mediated LDL was positively associated with rectal cancer (OR = 4.22, 95% CI: 1.21–14.73, *p* = 0.02) and hepatocellular carcinoma (OR = 82.99, 95% CI: 1.70–4062.70, *p* = 0.03). In the meta‐analysis, HMGCR‐mediated LDL was negatively associated with colon cancer (OR = 0.62, 95% CI: 0.49–0.78, *p* < 0.01) and hepatocellular carcinoma (OR = 0.06, 95% CI: 0.00–0.81, *p* = 0.03). PCSK9‐mediated LDL was positively associated with colon cancer (OR = 1.33, 95% CI: 1.10–1.16, *p* < 0.01) and negatively associated with rectal cancer (OR = 0.63, 95% CI: 0.53–0.76, *p* < 0.01) and hepatocellular carcinoma (OR = 0.63, 95% CI: 0.40–0.97, *p* = 0.04). NPC1L1‐mediated LDL was positively associated with rectal cancer (OR = 4.47, 95% CI: 2.31–8.65, *p* < 0.01).

For the subgroups in which replication analysis was not feasible (Table S4), we observed that HMGCR‐mediated LDL was negatively associated with lower third EC (OR = 0.16, 95% CI: 0.05–0.52, *p* < 0.01), colon in situ carcinoma (OR = 0.20, 95% CI: 0.05–0.82, *p* = 0.03), and anal canal cancer (OR = 0.16, 95% CI: 0.03–0.83, *p* = 0.03). PCSK9‐mediated LDL was positively associated with anal canal in situ carcinoma (OR = 11.86, 95% CI: 3.15–44.67, *p* < 0.01) and pancreatic head cancer (OR = 9.72, 95% CI: 4.25–22.27, *p* < 0.01). NPC1L1‐mediated LDL was negatively associated with esophageal adenocarcinoma (OR = 0.33, 95% CI: 0.15–0.73, *p* < 0.01).

### 3.3 Sensitivity Analysis

For the current positive MR results based on the IVW method, we observed heterogeneity in the association between HMGCR‐mediated LDL and hepatocellular carcinoma (*p* = 0.04) (Table S5). To assess horizontal pleiotropy, the results from MR‐Egger suggested the presence of pleiotropy for PCSK9‐mediated LDL in EC (*p* = 0.02), CRC (*p* < 0.01), and pancreatic head cancer (*p* = 0.01) (Tables S4 and S5). The results from MR‐PRESSO indicated that there was no horizontal pleiotropy in the current positive MR results. However, radial regression identified that some SNPs exhibited pleiotropy with the outcomes (Figures S4 and S5). In the positive MR analysis, we removed outliers to eliminate pleiotropy. For the genetic variants exhibiting pleiotropy (Table S6), we excluded rs2495495 and rs557435 in the analysis of PCSK9‐mediated LDL and EC (Figure S5A), excluded rs1159114 and rs11591147 in the analysis of PCSK9‐mediated LDL and colon cancer (Figure S5B,C), and excluded rs10788994, rs10788994, and rs11591147 in the analysis of PCSK9‐mediated LDL and pancreatic head cancer (Figure S5D). Subsequently, we repeated the MR analysis and observed the elimination of pleiotropy in the analysis of colon cancer and pancreatic head cancer. However, pleiotropy persisted in the association between PCSK9‐mediated LDL and EC (*p* = 0.02) (Table S7).

### 3.4 Colocalization Analysis

We initially conducted a colocalization analysis for HMGCR, PCSK9, and NPC1L1‐mediated LDL with outcomes. We found that NPC1L1‐mediated LDL shared the same causal variant with GC (PP.H4 = 0.89) (Table S8). This implied a shared genetic factor between the two phenotypes, and the relationship is bidirectional for both traits. Additionally, no shared loci variants were identified for HMGCR, PCSK9, and NPC1L1‐mediated LDL with other outcomes (Table S8). This suggested the stability of the current results and further supported the notion that the exposure influenced the outcome between the two traits. In the subgroup analysis, no explicit evidence of colocalization was found (Table S9). After removing outliers with horizontal pleiotropy, the colocalization results remained unchanged (Table S10).

In the colocalization analysis of eQTLs across different tissues (Table S11), after excluding outliers with horizontal pleiotropy, we found moderate evidence supporting the colocalization of NPC1L1 in subcutaneous adipose tissue (PP.H4 = 50.9) (Figure S6) and pancreas (PP.H4 = 67.9) (Figure S7) with GC in the Finn Gen study. These were identified as secondary target genes (Figure S6). HMGCR in visceral omentum adipose tissue (PP.H4 = 56.8) (Figure S8) received moderate evidence of support for colocalization with liver hepatocellular carcinoma in the Finn Gen study and was identified as a secondary target gene (34). Subgroup analysis did not provide clear colocalization evidence (Table S12).

### 3.5 SMR Analysis

SMR analysis results revealed (Table S13) that for the current positive results, decreased expression of the HMGCR gene in blood was associated with a reduced risk of LC (OR = 0.56, 95% CI: 0.36–0.88, *p* = 0.01) and PC (OR = 0.52, 95% CI: 0.28–0.96, *p* = 0.04). Decreased expression of the PCSK9 gene in blood was associated with a reduced risk of EC (OR = 0.57, 95% CI: 0.34–0.96, *p* = 0.03) and GC (OR = 0.40, 95% CI: 0.20–0.81, *p* = 0.01). Reduced expression of the NPC1L1 gene in subcutaneous adipose tissue was associated with a decreased risk of EC (OR = 0.73, 95% CI: 0.56–0.96, *p* = 0.02), but an increased risk of GC (OR = 1.33, 95% CI: 1.08–1.64, *p* < 0.01), CRC (OR = 1.11, 95% CI: 1.01–1.22, *p* = 0.03), and LC (OR = 1.46, 95% CI: 1.10–1.94, *p* = 0.01).

In subgroup analysis (Tables S13 and S14), for the current positive results, decreased expression of HMGCR in blood was associated with a reduced risk of colon cancer (OR = 0.63, 95% CI: 0.42–0.94, *p* = 0.02) and hepatocellular carcinoma (OR = 0.11, 95% CI: 0.02–0.68, *p* = 0.02). Decreased expression of PCSK9 in blood was associated with a reduced risk of rectal cancer (OR = 0.65, 95% CI: 0.45–0.95, *p* = 0.03), but an increased risk of pancreatic head cancer (OR = 3.11, 95% CI: 1.01–9.52, *p* = 0.046). Decreased expression of NPC1L1 in subcutaneous adipose tissue was associated with a reduced risk of anal canal cancer (OR = 0.44, 95% CI: 0.21–0.94, *p* = 0.04), but an increased risk of colon cancer (OR = 1.26, 95% CI: 1.01–1.57, *p* = 0.04).

The current SMR results are consistent with the direction of the MR results. In the repeated analysis after removing pleiotropy, we found that SMR results remain consistent with the MR results (Table S15). This further enhances the robustness of the findings.

We summarized the results of MR and SMR analyses, and these remained statistically significant in the meta‐analysis. Ultimately, decreased expression of HMGCR and LDL mediated by HMGCR was associated with a reduced risk of colon cancer, hepatocellular carcinoma, and PC. Reduced expression of PCSK9 and LDL mediated by PCSK9 was associated with a decreased risk of EC, GC, and rectal cancer. Increased expression of NPC1L1 and LDL mediated by NPC1L1 was positively associated with GC and CRC. In subgroup analysis, a positive association was observed only between PCSK9 expression and LDL mediated by PCSK9 and pancreatic head cancer.

After integrating the evidence from colocalization, the results only suggested a negative association between HMGCR expression or LDL mediated by HMGCR and the risk of hepatocellular carcinoma. Additionally, a positive association was observed between NPC1L1 expression or LDL mediated by NPC1L1 and the risk of gastric cancer.

### 3.6 SMR Sensitivity Analysis

Based on the HEIDI results, there was heterogeneity in the association between blood PCSK9 and rectal cancer (*p* = 0.01) (Table S13). Additionally, there was heterogeneity in the results for blood PCSK9 and anal cancer (*p* = 0.03) as well as anus cancer (*p* = 0.01). However, the SMR results did not reach statistical significance (*p* = 0.08, *p* = 0.61) (Table S13).

Combining the results of MR, SMR, and meta‐analysis, we further examined whether horizontal pleiotropy exists. This was done by investigating whether the expression of nearby genes significantly associated with top eQTL SNPs for HMGCR, PCSK9, and NPC1L1 is related to the outcomes. We identified a total of five genes, including HMGCR, three genes including PCSK9, and five genes including NPC1L1 (Table S14).

For the SMR analysis, we did not observe a significant association between the expression of nearby genes around the top eQTL SNPs for HMGCR, PCSK9, and NPC1L1 and the outcomes (Table S15). The HEIDI test did not reveal heterogeneity. This suggests that the current results further reduce the interference of pleiotropy.

## 4 Discussion

We comprehensively assessed the potential impact of genetic variants related to LDL mediated by HMGCR, PCSK9, and NPC1L1 on digestive system cancers. After constructing the genetic instruments, we discovered that the genetic variants associated with LDL mediated by HMGCR, PCSK9, and NPC1L1 have distinct effects on digestive system cancers, and these findings were validated through meta‐analysis across two independent GWAS datasets. After summarizing the results from SMR and colocalization analyses, the current MR results provide suggestive evidence for a negative correlation between LDL mediated by HMGCR and the risk of hepatocellular carcinoma. Strong evidence is also provided for a positive correlation between LDL mediated by NPC1L1 and the risk of GC. This suggests that HMGCR and NPC1L1 are promising drug targets for treating hepatocellular carcinoma and GC, and the current results are less likely to be influenced by pleiotropy.

Compared to developing new drugs, repurposing the existing drugs is more economical and time‐saving. In clinical practice, lipid‐lowering drugs can reduce LDL levels and significantly improve the prognosis of cardiovascular diseases. However, the efficacy of lipid‐lowering drugs in cancer patients remains controversial (Deng et al. 2022; Alsheikh‐Ali et al. 2007). In summary, the current differences can be attributed to the heterogeneity in study quality, patient samples, tumor characteristics, and potential confounding variables. Considering the complex dose–response relationship of the drug itself, prospective studies face challenges in distinguishing the impact of LDL reduction alone from the effects of drug use. In other words, are the observed benefits attributed to the direct action of the drug target, or are they a result of a greater extent of LDL reduction brought about by high‐dose lipid‐lowering medications ? Furthermore, digestive system cancers often exhibit high heterogeneity, further complicating the assessment of the actual effects of lipid‐lowering drugs on digestive system cancers.

In the current study, following detailed analysis and combining meta‐analysis results, the final evidence is concentrated on hepatocellular carcinoma. MR results suggest that reduced HMGCR expression or LDL mediated by HMGCR is associated with a decreased risk of hepatocellular carcinoma, providing suggestive evidence. The liver is a primary site for cholesterol synthesis, and much of the research on lipid‐lowering drugs has focused on the liver (Revilla et al. 2021). In a prospective trial, the statin drug group had a higher incidence of newly diagnosed cancers compared to the placebo group, and statin drugs increased liver enzyme levels (Shepherd et al. 2002). Elevated liver enzymes have been associated with an increased risk of LC (Huang et al. 2023). Furthermore, LDL is associated with cellular membrane cholesterol content (Thompson et al. 2003). Elevated cholesterol levels promote the translocation of CD44 to lipid rafts, inhibiting the progression of liver cancer (Yang et al. 2018). Relevant studies have shown that statin drugs, by depriving cells of cholesterol, counteract the inhibitory effect of dietary cholesterol on liver cancer (Zhao et al. 2019). Our meta‐analysis indicates a negative association between LDL mediated by PCSK9 and the risk of LC. Although this finding was not validated by SMR, it further suggests that genetic variations related to LDL and targeted by lipid‐lowering drugs may act as protective factors against LC. This aligns with the MR findings reported by Liang et al. (HMGCR‐LDL‐LC: OR = 0.201, *p* < 0.01) (Liang et al. 2023). It is noteworthy that LC encompasses various subtypes, such as hepatocellular carcinoma, cholangiocarcinoma, metastatic liver cancer, etc., with hepatocellular carcinoma being the most common form of primary liver cancer in adults. Liang et al.'s study did not specify the proportion of hepatocellular carcinoma in their analysis (Liang et al. 2023). Therefore, our research provides insights specifically into drug treatments for hepatocellular carcinoma.

For GC, our findings suggest that NPC1L1 expression or NPC1L1‐mediated LDL increases the risk of gastric cancer, providing strong evidence for the association. Prospective studies have indicated an association between high LDL levels and GC risk (Pih et al. 2021). Elevated LDL levels stimulate the growth of GC cells and suppress the immune system (Jung et al. 2008). Ezetimibe is the most widely used NPC1L1 inhibitor, effectively reducing LDL levels. Preclinical model data indicate that Ezetimibe inhibits cancer occurrence and progression through various mechanisms, including anti‐angiogenesis, anti‐inflammatory effects, and inhibition of tumor cell proliferation (Gu et al. 2022). In GC, the expression of NPC1L1 is associated with poor prognosis (Xin et al. 2022). However, there is limited research on the risk of GC with NPC1L1 inhibitors. In trials such as SEAS, SHARP, and IMPROVE‐IT, we found no significant difference in the incidence of GC between the Ezetimibe treatment group and the placebo group (*p* = 0.23, *p* = 0.60) (Nissen 2009). Furthermore, current clinical evidence does not suggest a clear association between Ezetimibe and GC risk. Given the lack of extensive evidence on Ezetimibe, genetic MR results provide additional support for the assessment. In addition, we conducted additional pleiotropy testing for nearby genes significantly associated with the top eQTL SNPs of NPC1L1. SMR did not identify any potential pleiotropic genes associated with gastric cancer, further strengthening the current results. Therefore, our study suggests that Ezetimibe may serve as a promising drug target for the treatment of GC.

Our study also revealed pleiotropic effects of genetic variations associated with LDL through target genes. Despite our efforts to mitigate the interference of pleiotropy, it remains unknown whether the genetic variations related to LDL are jointly regulated by different genes. Assessing the potential extent of this issue is challenging, as many genes exhibit pleiotropy, and the effects of variations may involve developmental compensation, where damage to one gene is compensated for by others during development (Benn et al. 2011). Due to the significant roles of epigenetic factors and gene expression in the pathogenesis of digestive system tumors, and given the consistent results between our SMR and MR analyses, this analysis suggests that LDL may be a potential mediator, exhibiting mechanisms similar to both carcinogenic and anticancer effects. This may explain the heterogeneous impact of lipid metabolism on the development of different cancers (Mahboobnia et al. 2021). Specifically, tumor cells rely on LDL for membrane lipid raft biosynthesis and signal transduction (Abramson 2011). Oxidized derivatives of LDL can also inhibit cancer cell proliferation through apoptosis (Gill et al. 2008; Lanterna et al. 2016), leading to contradictory results observed in epidemiological studies. Therefore, the current study suggests that the validity of drug target genes can be considered only when MR and SMR achieve consistent effects (Huang et al. 2021).

After integrating the results of MR and SMR, drug target genes are found to have a certain significance for colorectal cancer, pancreatic cancer, and esophageal cancer, with some supporting evidence from basic experiments (Gao et al. 2021; Ito et al. 2021; He et al. 2015). Unfortunately, the current results did not receive support from colocalization. However, this does not negate the potential therapeutic benefits of lipid‐lowering drugs for digestive system tumors, and these associations still warrant further exploration and validation. In addition, lipid metabolism therapy continues to play an important role in rare cancers. For example, in neuroendocrine neoplasms, lipid‐lowering drugs may possess antitumor potential, further highlighting that lipid profile monitoring could improve prognosis in cancer patients (Modica et al. 2022; Modica et al. 2024). Given the potential therapeutic significance of lipid‐lowering agents in rare cancers, our study, together with research on rare cancers, jointly suggests that lipid‐lowering drugs hold promising clinical prospects.

One strength of this study is the utilization of MR, SMR, and colocalization analysis, employing genetic instruments as proxies for drug exposure. This approach helps minimize confounding biases, and by additionally mitigating pleiotropy, it enhances causal inference. Furthermore, the use of GWAS data from different databases contributes to the robustness of the results. Another advantage is the restriction of our analysis to individuals of European ancestry, minimizing population stratification biases.

However, the current study has certain limitations. Firstly, MR analysis cannot fully substitute clinical trials in the real‐world context of drug interventions. Secondly, since genetic variations reflect the lifelong impact of changes in lipid levels on the risk of digestive system cancers, their effects may not directly compare to the short‐term impacts of lipid‐lowering drugs. Lastly, our study is confined to individuals of European ancestry, and these findings may not necessarily generalize to other ethnic populations.

## 5 Conclusion

In summary, this study utilized a comprehensive genetic approach to identify associations between lipid‐lowering drugs and the risk of digestive system tumors. The findings suggest an increased risk of hepatocellular carcinoma with HMGCR inhibitors, while NPC1L1 inhibitors are associated with a reduced risk of gastric cancer. However, these results warrant validation through large‐scale clinical trials, and further investigations are needed to explore the underlying mechanisms.

## Author Contributions

**Keru Ma:** conceptualization (equal), resources (equal). **Hao Wang:** conceptualization (equal), resources (equal). **Tianyu Chen:** conceptualization (equal), resources (equal). **Yubo Du:** conceptualization (equal), resources (equal). **Dongxu Yang:** conceptualization (equal), resources (equal). **Yue Li:** conceptualization (equal), resources (equal). **Dalin Li:** conceptualization (equal), data curation (equal).

## Ethics Statement

This research utilized publicly available summary statistics from published studies and consortia. All original studies had received approval from their respective ethical review boards, with participants providing informed consent. Since the study did not involve individual‐level data, no additional ethical review board approval was necessary.

## Consent

All authors have provided consent for publication.

## Conflicts of Interest

The authors declare no conflicts of interest.

## Supporting information


Data S1.

Table S1.
Table S2.
Table S3.
Table S4.
Table S5.
Table S6.
Table S7.
Table S8.
Table S9.
Table S10.
Table S11.
Table S12.
Table S13.
Table S14.
Table S15.

Figure S1.
Figure S2.
Figure S3.
Figure S4.
Figure S5.
Figure S6.
Figure S7.
Figure S8.

## Data Availability

All data were from open access GWAS large data, as detailed in Table S1.
