# Supplementary material for: Mendelian Randomization Assessment of the Genetic Effects of Lipid‐Lowering Drugs on Digestive System Cancers
Source: Food Sci Nutr. 2025 May 19;13(5):e70293. doi: 10.1002/fsn3.70293 (PMC12121511; doi:10.1002/fsn3.70293)

**Figure S1**: HMGCR-mediated LDL is associated with the risk of digestive system tumors. IVW- MR method was used to assess the association.


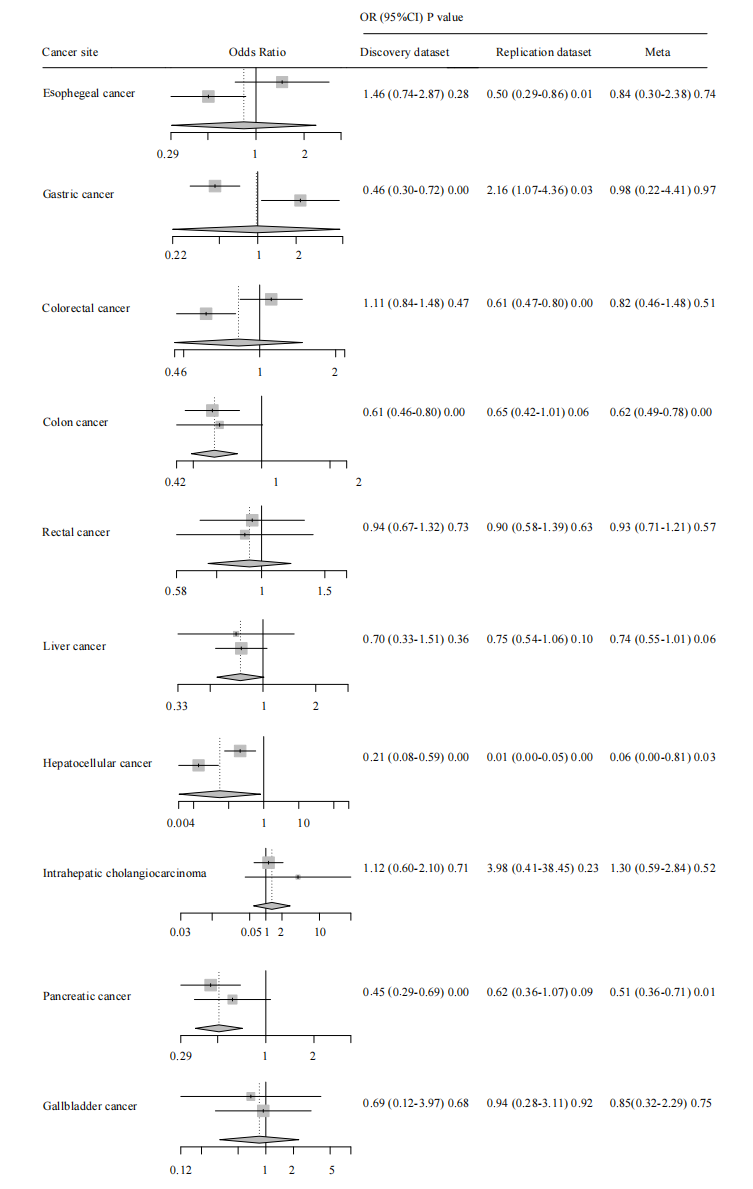


**Figure S2**: PCSK9-mediated LDL is associated with the risk of digestive system tumors. IVW- MR method was used to assess the association.
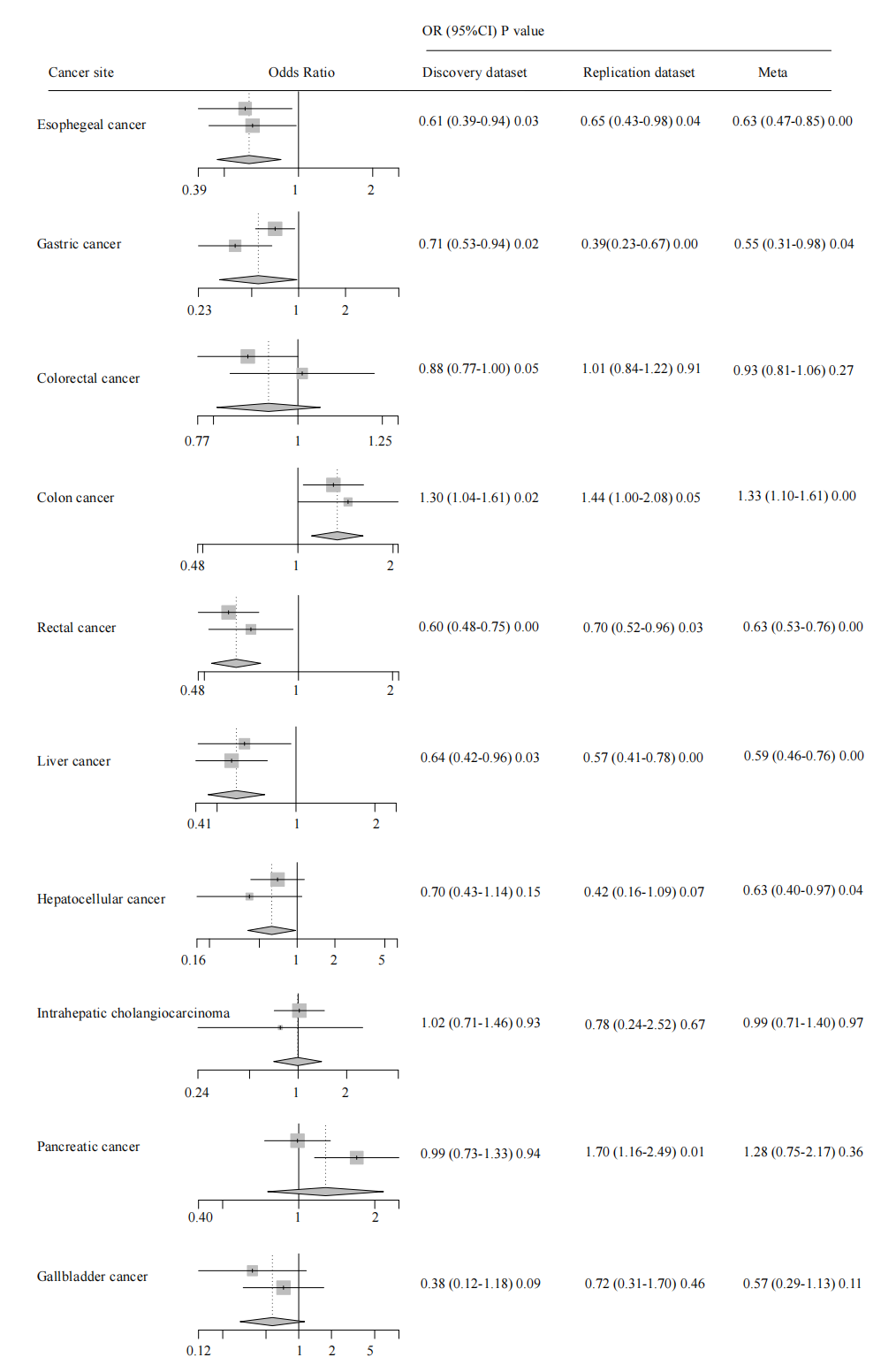


**Figure S3**: NPC1L1-mediated LDL is associated with the risk of digestive system tumors. IVW- MR method was used to assess the association.


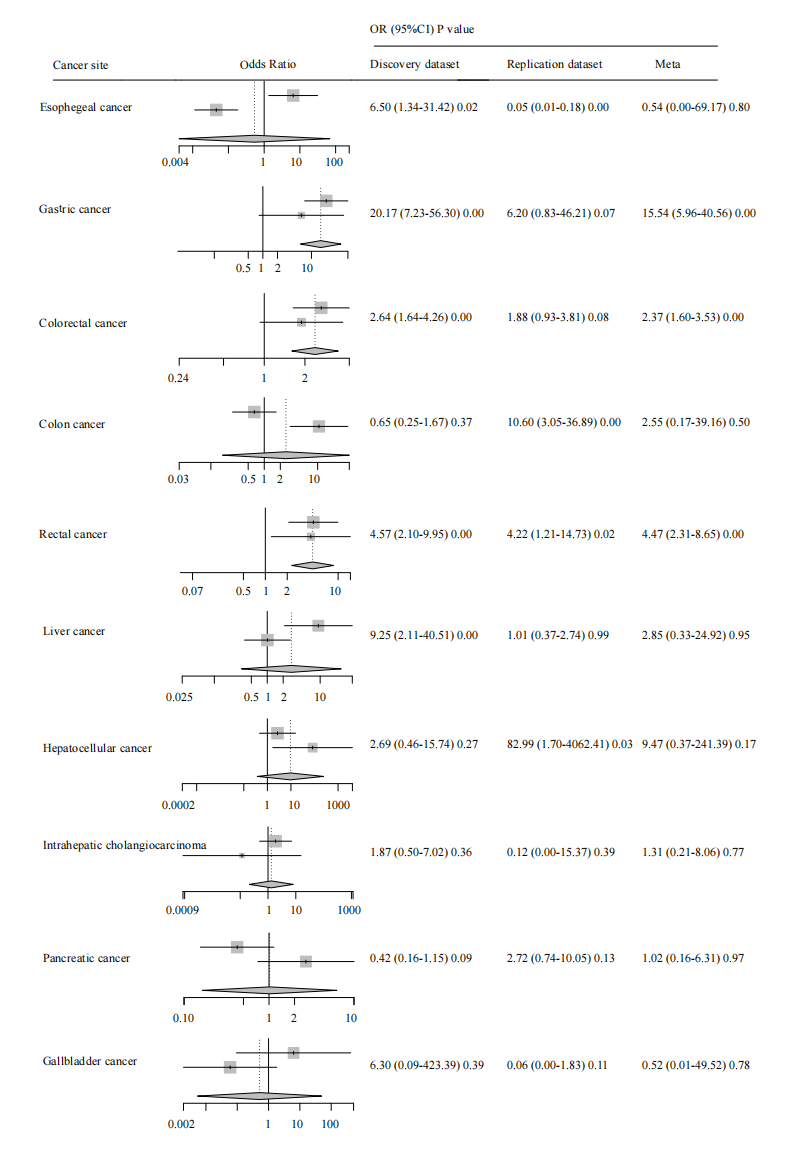


**Figure S4:**  Radial plots of the HMGCR-mediated LDL data produced using the RadialMR package. Radial lines joining each data point back to the origin.


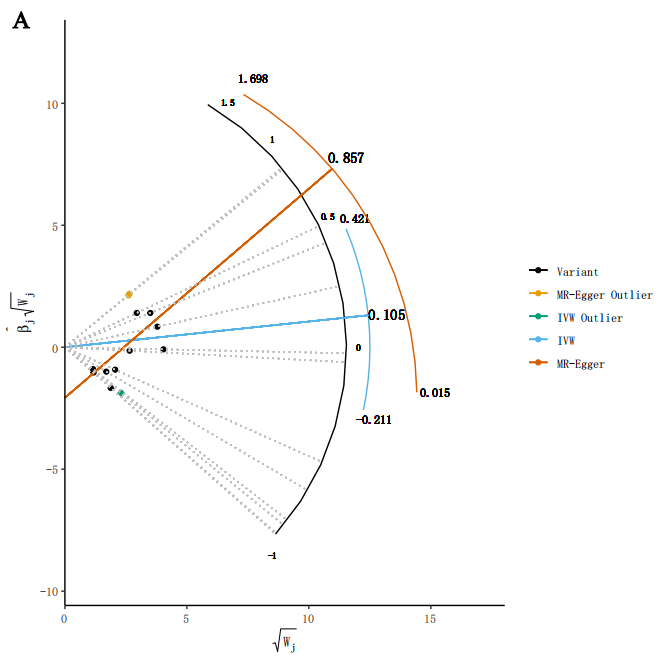


**S4 A:** Pleiotropic SNPs in colorectal in Finn Gen study.


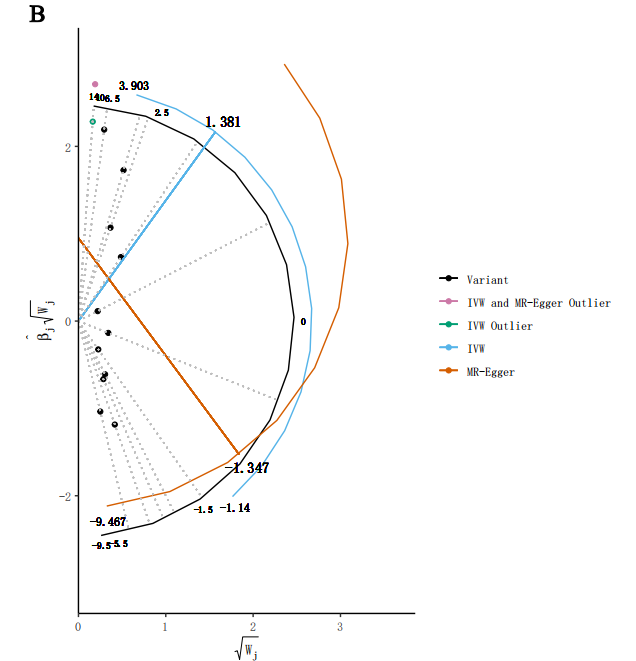


**S4 B:** Pleiotropic SNPs in intrahepatic cholangiocarcinoma in UK Biobank study.

**Figure S5:**  Radial plots of the PCSK9-mediated LDL data produced using the RadialMR package. Radial lines joining each data point back to the origin.


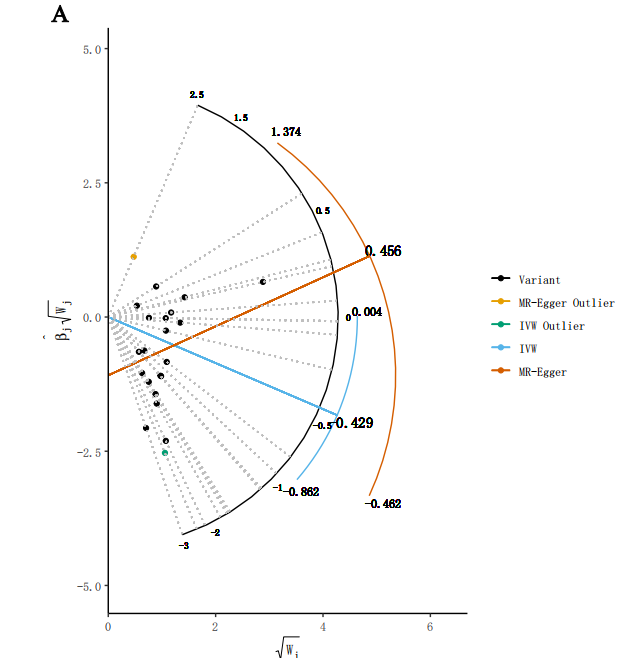


**S5 A:** Pleiotropic SNPs in esophageal cancer in pan-UK Biobank study.


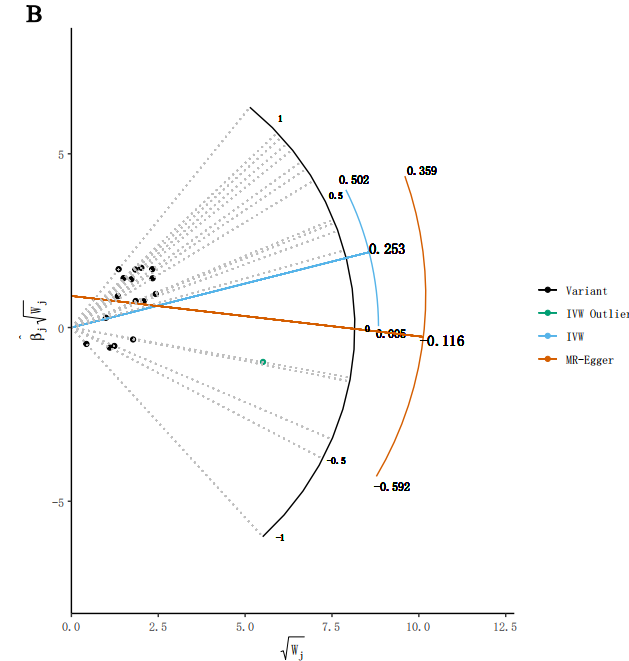


**S5 B:** Pleiotropic SNPs in colon cancer in UK Biobank study (GCST90011811).


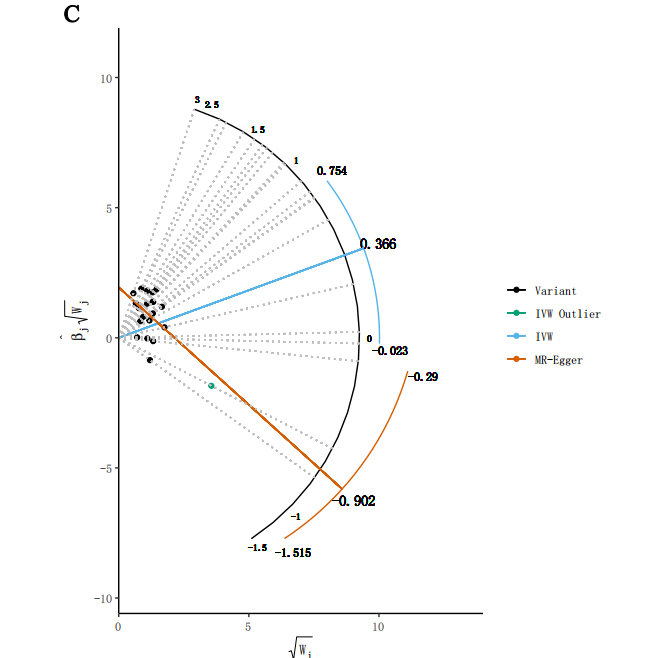


**S5 C:** Pleiotropic SNPs in colon cancer in UK Biobank study (GCST90041895).


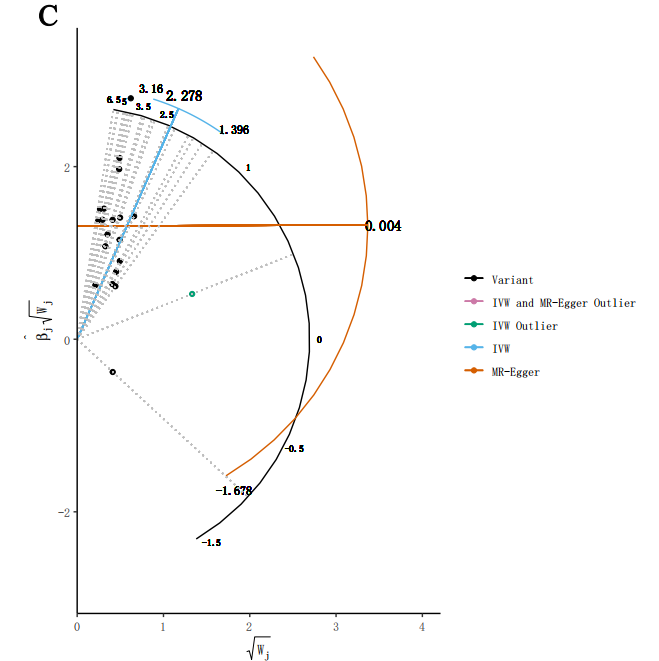


**S5 D:** Pleiotropic SNPs in pancreatic head cancer in UK Biobank study.

**Figure S6:** Colocalisation analysis of the cis-eQTL for NPC1L1 level in subcutaneous adipose tissue and gastric cancer risk.


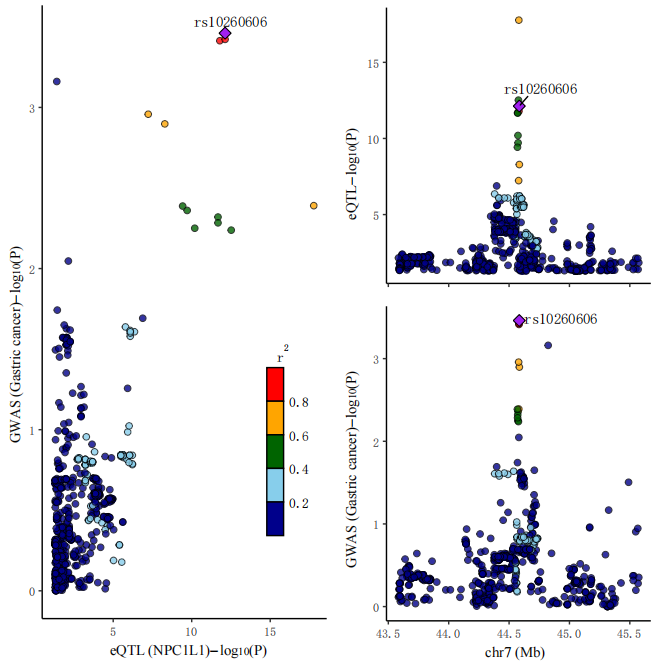


**Figure S7:** Colocalisation analysis of the cis-eQTL for NPC1L1 level in pancreatic tissue and gastric cancer risk.


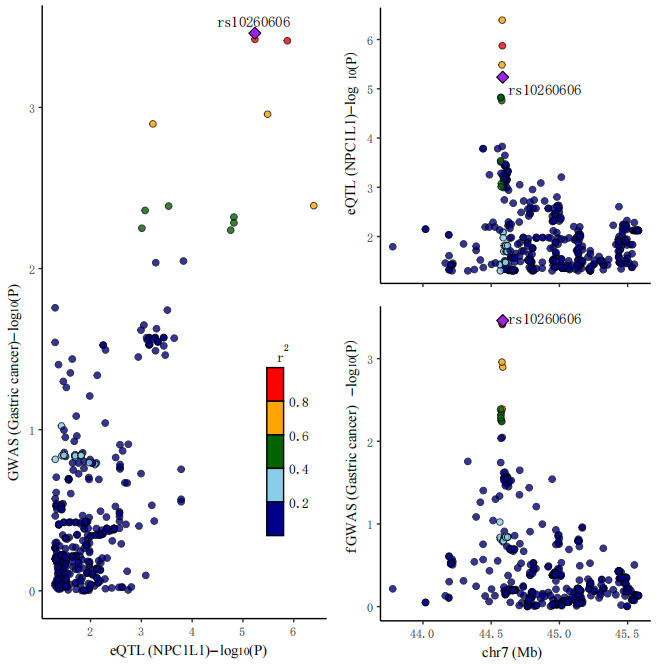


**Figure S8:** Colocalisation analysis of the cis-eQTL for HMGCR level in adipose visceral omentum tissue and hepatocellular cancer risk.


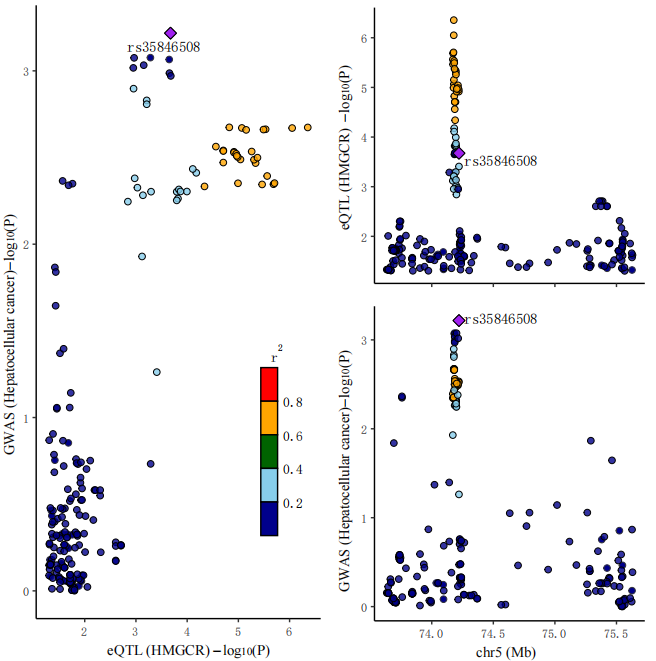

Supplement: Supplementary file 3 — Figure S1. Figure S2. Figure S3. Figure S4. Figure S5. Figure S6. Figure S7. Figure S8. [file FSN3-13-e70293-s003.docx]
